# Supplementary material for: Comparison of CRISPR and Marker-Based Methods for the Engineering of Phage T7
Source: Viruses. 2020 Feb 10;12(2):193. doi: 10.3390/v12020193 (PMC7077284; doi:10.3390/v12020193)
Supplement: Supplementary file 1 [file viruses-12-00193-s001.pdf]

Table S1. Bacterial strains used in this study.

Strain /Phage	Genotype	Reference
E. coli BW25113	BW25113 lacI+rrnBT14 ΔlacZWJ16 hsdR 514 ΔaraBADAH33 ΔrhaBADLD78 rph-1 Δ(araB–D)567 Δ(rhaD–B)568 ΔlacZ4787(::rrnB-3) hsdR514 rph-1	[1]
E. coli MG1655	K-12 F– λ– ilvG– rfb-50 rph-1	[2]
E. coli BW25113 ΔtrxA	F-, Δ(araDaraB) 567, ΔlacZ4787(::rrnB-3), λ-, rph-1, ΔtrxA732::kan, Δ(rhaDrhaB)568, hsdR514	[1]
E. coli BW25113 Δcmk	F-, Δ(araDaraB)567, ΔlacZ4787(::rrnB-3), λ-, Δcmk-734::kan, rph-1, Δ(rhaDrhaB)568, hsdR514	[1]
E. coli BL21-AI	BF– ompT gal dcm lon hsdSB(rB–mB–) [malB+]K-12(λS)	[3]
BL21(DE3)	B F^- ompT gal dcm lon $hsdS_B(r_B^- m_B^-)$ λ(DE3 [lacI lacUV5-T7p07 ind1 sam7 nin5]) $[malB^+]_{K-12}(\lambda^S)$	[4,5]

Table S2. Vectors used in this study.

Vector	Description Sequence/Relevant Information	Source/ Reference/Notes
**pWUR397**	Type I CRISPR - cas3 under T7 promoter, Kan^R	[6]
**pWUR400**	Type I cascade genes under T7 promoter, Str^R	[6]
**pAG_1**	pSMART, Amp^R, with TAATACGACTCACTATAGGGAGTTCCCCGCGCCAGCGGGGATAAACCGTGGTCTTCGACCAGTCTCGGAAGCTCAAAGGTCTGAAGACCAGAGTTCCCCGCGCCAGCGGGGCTAGTTATTGCTCAGCGG	This study/used as gRNAscr for type I CRISPR
**pAG_2**	pAG1 with gRNA TCCTTACGATTAATACAGACTATCGCTTTGCT	This study/ used as gRNA1 for type I CRISPR
**pAG_3**	pAG1 with gRNA AAATATTCACGCTAACGGGCGCCTTTACATGA	This study/used as gRNA2 for type I CRISPR
**pAG_4**	pAG1 with gRNA CGGTAACATCCAGTTAGTAGTAAACGGACAGA	This study/used as gRNA3 for type I CRISPR
**pAG_5**	pAG1 with gRNA TTACTCGACGTAACTCGATGGTCGTGTAGCCA	This study/ used as gRNA4 for type I CRISPR
**pAG_6**	pAG1 with gRNA TACAGTCATTGTTGTTATCTGACCCTCTACCA	This study/used as gRNA5 for type I CRISPR
**pAG_7**	pAG1 with gRNA CGTGGACTCAGGTGTGGTCTGGTAGTGCTGGC	This study/used as gRNA6 for type I CRISPR

**pAG_8**	pAG1 with gRNA TGTGGTCTGGTAGTGCTGGCGGTGGGGTAAGT	This study/ used as gRNA7 for type I CRISPR
**pAG_9**	pAG1 with gRNA ATCTCCGCTTCCGCAATATCTGGATTAAGTGT	This study/ used as gRNA8 for type I CRISPR
**pAG_10**	pAG1 with gRNA CTATGAAGTAGATTCCATCGGGGCCAGTACGG	This study/ used as gRNA9 for type I CRISPR
**pAG_11**	pAG1 with gRNA TACTGAACGACTGTCTGCAATATTCTTGAATC	This study/ used as gRNA10 for type I CRISPR
**pCas_9**	Cmp^R, with tgagaccagtctcggaagctcaaaggtctc	[61]/used as gRNAscr for type II CRISPR
**pAG_12**	pCas_9 with gRNA AAGTGTGACTGTTTCACAGG	This study/ This study/ used as gRNA1 for type II CRISPR
**pAG_13**	pCas_9 with gRNA AGGCGTGGACTCAGGTGTGG	This study/ used as gRNA2 for type II CRISPR
**pAG_14**	pCas_9 with gRNA AGTGTGCCAACAACTCTTGG	This study/ used as gRNA3 for type II CRISPR
**pAG_15**	pCas_9 with gRNA TTCCGCTGCGCATCAATCTG	This study/ used as gRNA4 for type II CRISPR
**pAG_16**	pCas_9 with gRNA ACGCTACGAACACAAAGCAG	This study/ used as gRNA5 for type II CRISPR
**pAG_17**	pCas_9 with gRNA CAGCATCCGCTAACTCTGCTC	This study/ used as gRNA6 for type II CRISPR
**pAG_18**	pCas_9 with gRNA TACAGTTCCGTAATGAGGCT	This study/ used as gRNA7 for type II CRISPR
**pAG_19**	pCas_9 with gRNA GGTAAGTGTGACTGTTTCAC	This study/ used as gRNA8 for type II CRISPR
**pAG_20**	pCas_9 with gRNA GATCTCCGCTTCCGCAATAT	This study/ used as gRNA9 for type II CRISPR
**pAG_21**	pCas_9 with gRNA CTTCCGCAATATCTGGATTA	This study/ used as gRNA10 for type II CRISPR
**pAG_22**	pCas_9 with gRNA AATACACTCCAACGGTCTCG	This study/ used as gRNA11 for type II CRISPR
**pSB6A1**	Amp^R	Registry of Standard Biological parts
**pAG_23**	pSB6A1 with HR1*, 513-1146 (*mtd), RBS (B0034), trxA (full sequence)*, HR2**	This study
**pAG_24**	pSB6A1 with HR1*, 513-1146 (*mtd), RBS (B0034), trxA (full sequence)*, HR2**	This study
**pAG_25**	pSB6A1 with HR1*, 513-1146 (*mtd), RBS (B0034), trxA (full sequence)*, HR2**	This study
**pAG_26**	pSB6A1 with HR1*, 513-1146 (*mtd), RBS (B0034), trxA (full sequence)*, HR2**	This study

**pAG_27**	pSB6A1 with HR1*, 513-1146 (*mtd), RBS (B0034), trxA (full sequence)*, HR2**	This study
**pAG_28**	pSB6A1 with HR1*, 513-1146 (*mtd), RBS (B0034), trxA (full sequence)*, HR2**	This study
**pAG_29**	pSB6A1 with HR1*, 513-1146 (*mtd), RBS (B0034), trxA (full sequence)*, HR2**	This study
**pAG_30**	pSB6A1 with full *g17* under T7 promoter	This study
**pAG_31**	pSEVA551 with codon optimised full *g17* under T7 promoter	This study
**pAG_32**	pSB6A1 with HR1*, 1455-1707 (Yep-phi, *g17*), *RBS (B0034), trxA (full sequence)*, HR2**	This study

HR1* gccgttgtggccactgatggtaatattcaaggtactaagtggggaggtaaatggctggatgcttacctacgtgacagcttcgttgcgaag

HR2** ttggtaaatcacaaggaaagacgtgtagtccacggatggactctcaaggaggtacaaggtgctatcattagactttaacaacgaattgat

Table S3. Primers used in this study.

Primer Sequence 5′ to 3′	Use
ACCGTCCTTACGATTAATACAGACTATCGCTTTGCT	Forward primer to generate insert for pAG_2
ACTCAGCAAAGCGATAGTCTGTATTAATCGTAAGGA	Reverse primer to generate insert for pAG_2
ACCGAAATATTCACGCTAACGGGCGCCTTTACATGA	Forward primer to generate insert for pAG_3
ACTCTCATGTAAAGGCGCCCGTTAGCGTGAATATTT	Reverse primer to generate insert for pAG_3
ACCGCGGTAACATCCAGTTAGTAGTAAACGGACAGA	Forward primer to generate insert for pAG_4
ACTCTCTGTCCGTTTACTACTAACTGGATGTTACCG	Reverse primer to generate insert for pAG_4
ACCGTTACTCGACGTAACTCGATGGTCGTGTAGCCA	Forward primer to generate insert for pAG_5
ACTCTGGCTACACGACCATCGAGTTACGTCGAGTAA	Reverse primer to generate insert for pAG_5
ACCGTACAGTCATTGTTGTTATCTGACCCTCTACCA	Forward primer to generate insert for pAG_6
ACTCTGGTAGAGGGTCAGATAACAACAATGACTGTA	Reverse primer to generate insert for pAG_6
ACCGCGTGGACTCAGGTGTGGTCTGGTAGTGCTGGC	Forward primer to generate insert for pAG_7
ACTCGCCAGCACTACCAGACCACACCTGAGTCCACG	Reverse primer to generate insert for pAG_7
ACCGTGTGGTCTGGTAGTGCTGGCGGTGGGGTAAGT	Forward primer to generate insert for pAG_8
ACTCACTTACCCCACCGCCAGCACTACCAGACCACA	Reverse primer to generate insert for pAG_8
ACCGATCTCCGCTTCCGCAATATCTGGATTAAGTGT	Forward primer to generate insert for pAG_9
ACTCACACTTAATCCAGATATTGCGGAAGCGGAGAT	Reverse primer to generate insert for pAG_9
ACCGCTATGAAGTAGATTCCATCGGGGCCAGTACGG	Forward primer to generate insert for pAG_10
ACTCCCGTACTGGCCCCGATGGAATCTACTTCATAG	Reverse primer to generate insert for pAG_10
ACCGTACTGAACGACTGTCTGCAATATTCTTGAATC	Forward primer to generate insert for pAG_11
ACTCGATTCAAGAATATTGCAGACAGTCGTTCAGTA	Reverse primer to generate insert for pAG_11
AAACAAGTGTGACTGTTTCACAGG	Forward primer to generate insert for pAG_12

AAAACCTGTGAAACAGTCACACTT	Reverse primer to generate insert for pAG_12
AAACAGGCGTGGACTCAGGTGTGG	Forward primer to generate insert for pAG_13
AAAACCACACCTGAGTCCACGCCT	Reverse primer to generate insert for pAG_13
AAACAGTGTGCCAACAACTCTTGG	Forward primer to generate insert for pAG_14
AAAACCAAGAGTTGTTGGCACACT	Reverse primer to generate insert for pAG_14
AAACGTTCCGCTGCGCATCAATCTG	Forward primer to generate insert for pAG_15
AAAACAGATTGATGCGCAGCGGAAC	Reverse primer to generate insert for pAG_15
AAACGACGCTACGAACACAAAGCAG	Forward primer to generate insert for pAG_16
AAAACTGCTTTGTGTTCGTAGCGTC	Reverse primer to generate insert for pAG_16
AAACGAGCAGAGTTAGCGGATGCTG	Forward primer to generate insert for pAG_17
AAAACAGCATCCGCTAACTCTGCTC	Reverse primer to generate insert for pAG_17
AAACGAGCCTCATTACGGAACTGTA	Forward primer to generate insert for pAG_18
AAAATACAGTTCCGTAATGAGGCTC	Reverse primer to generate insert for pAG_18
AAACGGTAAGTGTGACTGTTTCAC	Forward primer to generate insert for pAG_19
AAAAGTGAAACAGTCACACTTACC	Reverse primer to generate insert for pAG_19
AAACGATCTCCGCTTCCGCAATATC	Forward primer to generate insert for pAG_20
AAAAGATATTGCGGAAGCGGAGATC	Reverse primer to generate insert for pAG_20
AAACGTAATCCAGATATTGCGGAAG	Forward primer to generate insert for pAG_21
AAAACTTCCGCAATATCTGGATTAC	Reverse primer to generate insert for pAG_21
AAACGCGAGACCGTTGGAGTGTATT	Forward primer to generate insert for pAG_22
AAAAAATACACTCCAACGGTCTCGC	Reverse primer to generate insert for pAG_22
GTGACAGCTTCGTTGCGAAGATCAAGTTTCGCCCGGCTGC	For pAG_26 construct Gibson assembly; *mtd* g-block forward
TGACCTCCTTAAAGTAAATCACAAAAAACCCCTAGCCGCC	*mtd* g-block reverse
ACGCCAACCTGGCTTAATGATTGGTAAATCACAAGGAAAGACG	pSB6A1 (Yep-phi) *p17*g-block g-block forward
GCAGCCGGGCGAAACTTGATCTTCGCAACGAAGCTGTCAC	pSB6A1 (Yep-phi) *p17*g-block g-block reverse
GGCGGCTAGGGGTTTTTTGTGATTTACTTTAAGGAGGTCAAATGAG	*trxA* g-block forward
CTTTCCTTGTGATTTACCAATCATTAAGCCAGGTTGGCGT	*trxA* g block reverse

GTGACAGCTTCGTTGCGAAGAACGAATACAGCCTGTGGGA	For pAG_25 construct Gibson assembly; *mtd* g-block forward
TGACCTCCTTAAAGTAAATCACAAAAAACCCCTAGCCGCC	*mtd* g-block reverse
ACGCCAACCTGGCTTAATGATTGGTAAATCACAAGGAAAGACG	pSB6A1 (Yep-phi) *p17*g-block g-block forward
TCCCACAGGCTGTATTCGTTCTTCGCAACGAAGCTGTCAC	pSB6A1 (Yep-phi) *p17*g-block g-block reverse
GGCGGCTAGGGGTTTTTTGTGATTTACTTTAAGGAGGTCAAATGAG	*trxA* g-block forward
CTTTCCTTGTGATTTACCAATCATTAAGCCAGGTTGGCGT	*trxA* g-block reverse
GTGACAGCTTCGTTGCGAAGAACGAATACAGCCTGTGGGA	For pAG_24 construct Gibson assembly; *mtd* g-block forward
TGACCTCCTTAAAGTAAATCACAAAAAACCCCTAGCCGCC	to amplify *mtd* g-block reverse
ACGCCAACCTGGCTTAATGATTGGTAAATCACAAGGAAAGACG	pSB6A1 (Yep-phi) *p17*g-block g-block forward
TCGGCCTTGATGAAAGCCGTCTTCGCAACGAAGCTGTCAC	pSB6A1 (Yep-phi) *p17*g-block g-block reverse
GGCGGCTAGGGGTTTTTTGTGATTTACTTTAAGGAGGTCAAATGAG	*trxA* g-block forward
CTTTCCTTGTGATTTACCAATCATTAAGCCAGGTTGGCGT	*trxA* g-block reverse
ACCACCTGATTCTTGAGTAGCGGGGCCGAAAGGCCCCGCC	For pAG_27 construct Gibson assembly; pAG_24 forward
ACCAGATCGTGCCGCGCCAGCTTCGCAACGAAGCTGTCACGTAGGTAAGC	pAG24 reverse
GTGACAGCTTCGTTGCGAAGCTGGCGCGGCACGATCTGGT	*mtd* g-block forward
GGCGGGGCCTTTCGGCCCCGCTACTCAAGAATCAGGTGGTCACAGACG	*mtd* g-block reverse
GTGACAGCTTCGTTGCGAAGAAGTTTCGCCCGGCTGCGCT	For pAG_23 construct assembly; *mtd* g-block forward
TGACCTCCTTAAAGTAAATCCGGTTGCCTTGGCGGGGCCT	*mtd* g-block reverse
ACGCCAACCTGGCTTAATGATTGGTAAATCACAAGGAAAGACG	pSB6A1 (Yep-phi) *p17*g-block g-block forward
AGCGCAGCCGGGCGAAACTTCTTCGCAACGAAGCTGTCAC	pSB6A1 (Yep-phi) *p17*g-block g-block reverse
AGGCCCCGCCAAGGCAACCGGATTTACTTTAAGGAGGTCAAATGAG	*trxA* g-block forward
CTTTCCTTGTGATTTACCAATCATTAAGCCAGGTTGGCGT	*trxA* g-block reverse
ACCACCTGATTCTTGAGTAGCGGGGCCGAAAGGCCCCGCC	For pAG_28 construct Gibson assembly; pAG_24 forward

ATGAAAGCCGTTTTCACCAGCTTCGCAACGAAGCTGTCACGTAGGTAAGC	pAG_24 reverse
GTGACAGCTTCGTTGCGAAGCTGGTGAAAACGGCTTTCAT	*mtd* g-block forward
GGCGGGGCCTTTCGGCCCCGCTACTCAAGAATCAGGTGGT	*mtd* g-block reverse
GGCCCTTTCGTCTTCAAGAATGTTAACTTGAGGGAGCGTA	*trxA* g-block forward
CACGATGCGTCCGGCGTAGATCAAATCAATTCGTTGTTAAAGTC	*trxA* g-block reverse
TTAACAACGAATTGATTTGATCTACGCCGGACGCATCGTG	pSB6A1 forward
ACGCTCCCTCAAGTTAACATTCTTGAAGACGAAAGGGCCTC	pSB6A1 reverse
GGCCTGCAGGAGTCACTACTAGTAGCGGCCGCTG	Gibson primers to generate pAG_32
CGCCGCGCGCCCCGAAGTTAGTTTCGAACTAAGATTTGC	
TCTTAGTTCGAAACTAACTTCGGGGCGCGCGGCG	
CAGCGGCCGCTACTAGTAGTGACTCCTGCAGGCCTTAATCAATTCGTTGTT	
TGGACTCACAAAGAAAAAACGCCCGGTGTGCAAGACCGAGCGTTCTGAACAATTACTCGTTCTCCACCATGATTGC	Gibson primers to generate pAG_30, *g17* forward
CTCACTATAGGGAGAACTAGAGAAAGAGGAGAAATACTAGATGGCTAACGTAATTAAAACCGTTTTGAC	*g17* reverse
GTCAAAACGGTTTTAATTACGTTAGCCATCTAGTATTTCTCCTCTTTCTCTAGTTCTCCCTATAGTGAG	pSB6A1 forward
GCAATCATGGTGGAGAACGAGTAATTGTTCAGAACGCTCGGTCTTGCACACCGGGCGTTTTTTCTTTGTGAGTCCA	pSB6A1 reverse
TCGGCTGGCTTTGTGGCTAACG	T7 sequencing, before *g17*
ACCTCCTTGAGAGTCCATCCGTGG	T7 sequencing, after *g17*

Table S4. Summary of phage mutants generated in this study.

T7 and BPP-1 tail fibre fusions	Gp17/*g17* aa/bp	Mtd/*mtd* aa/bp	Phage mutants generated*	HR vectors used for phage mutants
1	1 - 466/1-1398	171 – 382/513 – 1146	phAG_1	pAG23
2	1 - 466/1-1398	55 – 382/165 – 1146	phAG_2	pAG24
3	1 - 466/1-1398	163 – 382/489 – 1146	phAG_3	pAG25
4	1 - 466/1-1398	170 – 382/510 – 1146	phAG_4	pAG26
5	1 - 466/1-1398	47 – 382/141 – 1146	phAG_5	pAG27
6	1 - 466/1-1398	52 – 382/156 – 1146	phAG_6	pAG28
7	N/A	N/A	phAG_7**	pAG29
8	1 - 466/1-1398	(Yep-phi/gp17) 485-569/1455-1707	phPM	pAG32

*each mutant contains full sequence *trxA* after *mtd*/Yep-phi *g17* sequence insert.

**phAG_7 has g17 replaced with full sequence of *trxA.*

Table S5. Efficiency of generating phage T7 mutants. *In trans* method tail fiber mutant efficiency represented as plaque PCR screening output.

Phage Mutant	Plaques screened	Successful mutants (%)
**phAG_1**	**30**	**100**
**phAG_2**	**30**	**100**
**phAG_3**	**30**	**100**
**phAG_4**	**15**	**100**
**phAG_5**	**15**	**100**
**phAG_6**	**10**	**100**
**phAG_7**	**10**	**100**

Table S6. Single nucleotide variations detected in phage T7 mutants

Phage	SNV Position	Wild type	Mutant	Amino Acid change
phAG_3	24876	A	G	K->R
phAG_4	742	T	C	intergenic
phAG_4	1897	C	.	D->E
phAG_4	1899	A	C	Q->K

phAG_4	1900	G	A	Q->K
phAG_4	19560	T	G	V->G
phPM	26041	A	G	I>M

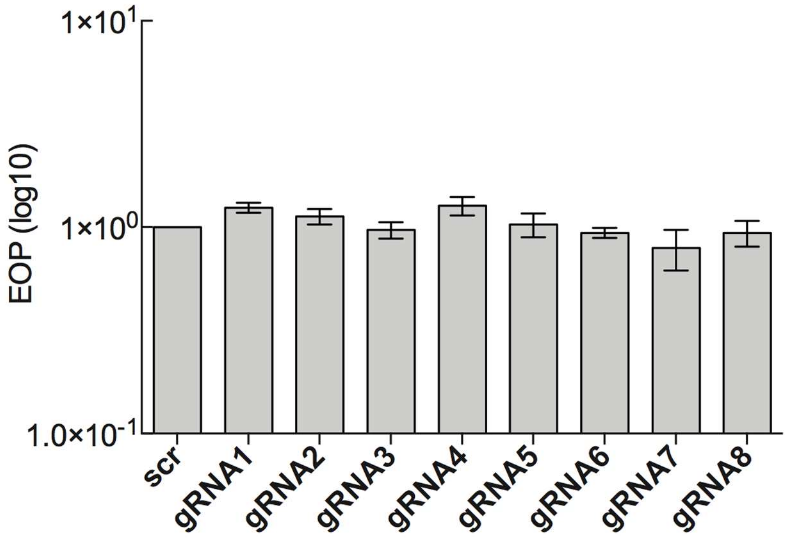

Figure S1. Efficiency of gRNAs only vectors of type I CRISPR-Cas system. Efficiency of plating for T7 against *E. coli* BW25113 containing type I CRISPR gRNAs only. The T7 efficiency of plating was determined with respect to a reference *E. coli* BW25113/pAG1 strain. EOP data is presented as the mean of three independent experiments. The concentration of the phage stock added 2×10^9 PFU/ml.

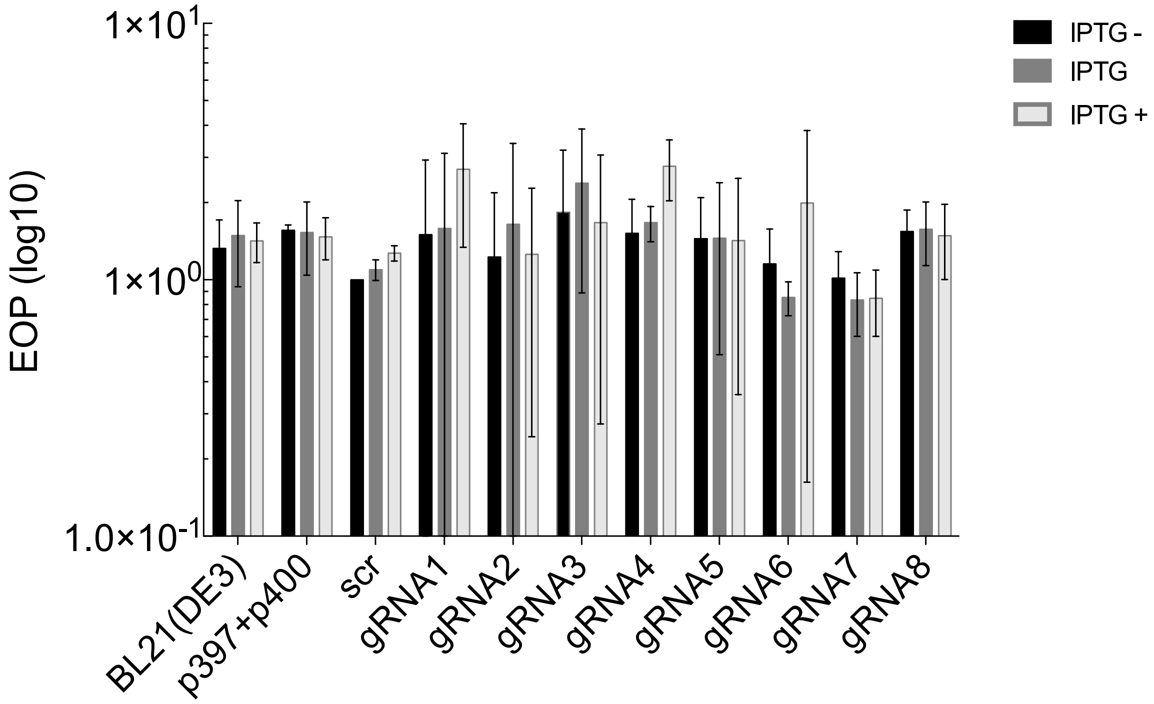

Figure S2. Efficiency of type I CRISPR-Cas system with and without IPTG induction. Efficiency of plating for T7 against *E. coli* BL21 (DE3) containing pWUR400 and pWUR397, and one of the nine gRNAs. The T7 efficiency of plating was determined with respect to a reference uninduced *E. coli* BL21(DE3) containing gRNA(scr). IPTG (-) group has no IPTG added. IPTG group had IPTG added only to the bacterial cultures. IPTG (+) group had IPTG added to the bacterial cultures as well as the top agar. EOP data is presented as the mean of three independent experiments. The concentration of the phage stock added 10^{10} PFU/ml.

E. coli BL21(DE3) strain containing the Type I CRISPR-Cas plasmids were grown in liquid LB medium to OD_{600} = 0.3-0.4. Expression of the CRISPR-Cas genes was induced by the addition of IPTG to a final concentration of 0.1 mM and the cultures incubated at 37°C for 1 hour. For each of the induced cultures, 1 mL was combined with 8 mL of 0.7% LB agar, with and without 0.1 mM IPTG, and plated onto 1.5% LB agar plates before being further incubated at 37°C for 1 hour. T7 was used to perform spot assays followed by incubation at 37°C overnight to calculate efficiency of plating.

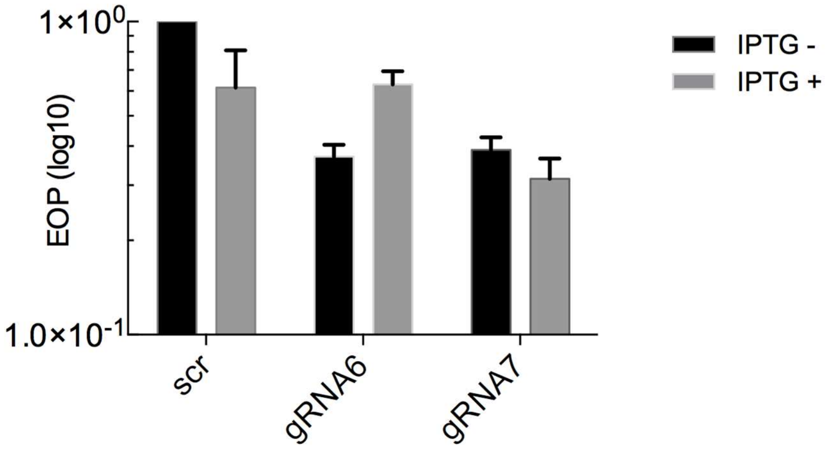

Figure S3. Efficiency of type I CRISPR-Cas system with and without IPTG induction. Efficiency of plating for T7 against *E. coli* BW25113 containing pWUR400 and pWUR397 and one of the three gRNA vectors pAG1 (gRNAscr), pAG7 (gRNA6) and pAG8 (gRNA7). The T7 efficiency of plating was determined with respect to a reference undinduced *E. coli* BW25113/pAG1 strain. EOP data is presented as the mean of three independent experiments. The concentration of the phage stock added $2x10^9$ PFU/ml.

E. coli BL21-AI strain containing Type I CRISPR-Cas plasmids were grown in liquid LB medium to OD_{600} = 0.3-0.4. Expression of the CRISPR-Cas genes was induced by the addition of IPTG to a final concentration of 0.1 mM and the cultures incubated at 37°C for 1 hour. Each of the induced cultures were then used to carryout plaque assays, followed by incubation at 37°C overnight to calculate efficiency of plating.

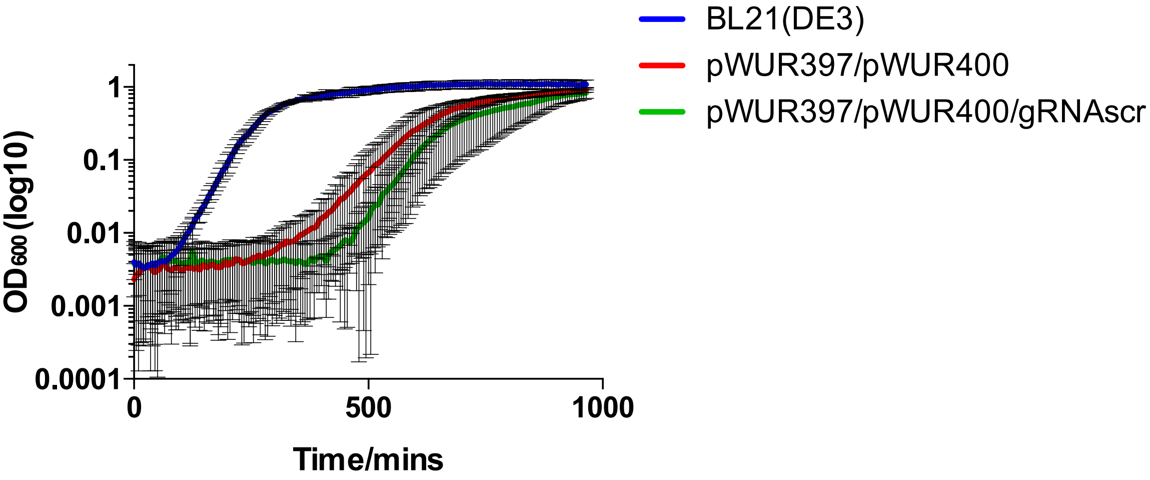

Figure S4. Growth of BL21(DE3) with and without type I CRISPR vectors. The data is presented for twenty-four technical replicates. The growth rate for each of the strains was determined. The growth rate for BL21(DE3) was 27.97 (+/- 0.46) mins. The growth rate for BL21(DE3) containing pWUR397/pWUR400 vectors was 38.92 (+/- 0.80) mins. The growth rate for BL21(DE3) containing pWUR397/pWUR400/gRNAscr was 96.14 (+/- 92.68) mins

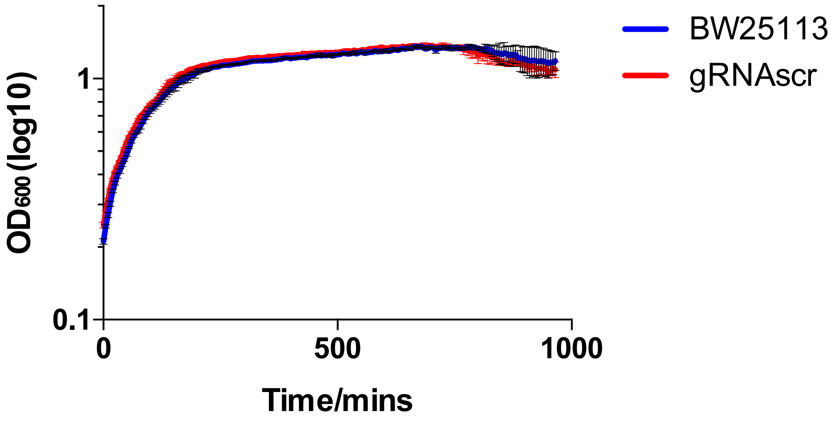

Figure S5. Growth of BW25113 with and without type II CRISPR vector. The data is presented for three technical replicates.

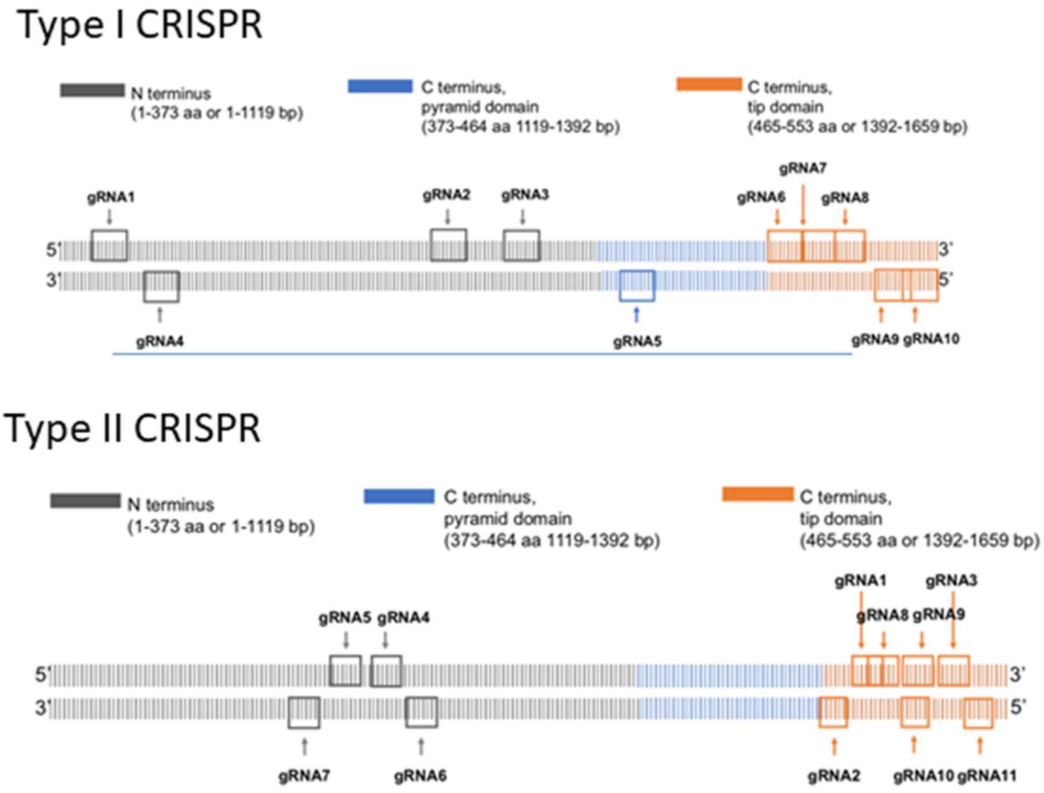

Figure S6. Representation of the distribution of gRNAs on *g17* designed for type I and type II CRISPRs.

(BPP-1) *mtd* g-block:

ATGAGTACCGCAGTCCAATTCCGAGGTGGAACGACCGCCCAGCACGCAACGTTCACGGGCGCCGCC
CGTGAGATTACCGTCGATACCGACAAGAACACGGTCGTTGTGCATGACGGTGCTACCGCTGGCGGCTT
CCCCCTGGCGCGGCACGATCTGGTGAAAACGGCTTTCATCAAGGCCGACAAGTCGGCCGTCGCCTTCA
CGCGCACCGGCAACGCAACGGCCAGCATCAAGGCTGGCACCATCGTGGAGGTCAACGGCAAGCTGGT
GCAGTTCACCGCCGACACGGCCATCACCATGCCGGCGCTGACGGCCGGCACCGACTACGCCATCTAC
GTCTGCGACGATGGCACGGTGCGCGCCGATTCCAACTTTTCGGCGCCCACTGGCTACACCTCGACCAC
GGCGCGCAAGGTGGGCGGCTTCCACTATGCGCCGGGAAGCAACGCTGCAGCGCAGGCTGGTGGAAAC
ACCACGGCGCAGATCAACGAATACAGCCTGTGGGACATCAAGTTTCGCCCGGCTGCGCTCGACCCGC
GCGGCATGACGCTGGTTGCCGGCGCGTTTTGGGCAGACATCTATCTGCTAGGCGTCAACCACCTGACC
GATGGCACCAGCAAATACAACGTGACAATTGCAGATGGTAGTGCATCACCTAAGAAATCTACCAAGT
TCGGTGGAGACGGCAGCGCGGCCTACAGTGACGGAGCTTGGTACAACTTCGCTGAGGTCATGACTCAT
CACGGTAAGCGCCTGCCTAACTACAACGAATTCCAGGCGCTGGCTTTCGGCACGACCGAGGCTACGTC
CAGCGGCGGCACCGACGTGCCCACCACCGGCGTGAACGGCACGGGCGCAACGAGCGCGTGGAACAT
CTTCACGTCCAAGTGGGGCGTTGTGCAGGCGTCCGGTTGCTTGTGGACGTGGGGTAACGAGTTCGGCG

GCGTGAATGGCGCATCCGAATACACGGCCAACACTGGCGGCAGAGGATCGGTGTACGCCCAGCCCGC
TGCTGCGCTATTCGGCGGCGCCTGGAACGGCACGTCGCTCTCGGGTTCTCGCGCTGCGCTCTGGTACAG
CGGGCCGTCGTTCTCGTTCGCGTTCTTCGGGGCGCGCGGCGTCTGTGACCACCTGATTCTTGAGTAGCG
GGGCCGAAAGGCCCCGCCAAGGCAACCGTACTCGAACCCCTAGCCCGCTCTTATCGGGCGGCTAGGG
GTTTTTTGT

HR1-*trxA*-HR2 g-block:

TGTTAACTTGAGGGAGCGTAGGAAATAATACGACTCACTATAGGGAGAGGCGAAATAATCTTCTCCCT
GTAGTCTCTTAGATTTACTTTAAGGAGGTCAAATGAGCGATAAAATCATTCACCTGACCGATGACTCTT
TTGATACCGACGTGCTGAAAGCTGATGGTGCAATTCTGGTTGATTTCTGGGCAGAGTGGTGCGGCCCTT
GCAAAATGATCGCTCCAATCCTGGACGAAATTGCGGACGAATATCAGGGTAAGCTGACTGTGGCCAA
ACTGAACATTGACCAGAACCCTGGCACCGCACCGAAATACGGTATCCGTGGCATCCCAACTCTGCTGC
TGTTCAAAAACGGTGAAGTGGCAGCAACCAAAGTAGGCGCTCTGTCTAAAGGCCAACTGAAAGAGTT
CCTGGACGCCAACCTGGCTTAATGATTGGTAAATCACAAGGAAAGACGTGTAGTCCACGGATGGACT
CTCAAGGAGGTACAAGGTGCTATCATTAGACTTTAACAACGAATTGATTTGA

(Yep-phi) *g17* g-block:

GCCGTTGTGGCCACTGATGGTAATATTCAAGGTACTAAGTGGGGAGGTAAATGGCTGGATGCTTACC
TACGTGACAGCTTCGTTGCGAAGAGCTCTGGTTGGACTGAGGTATGGCAAGGCTCTGCTGGTGGTGGT
GTTTCAGTAAGCCTCTCACAGGATGTCCGCTGGAGAACTATCTGGATTCTAGCTAATAATGGCATGTGT
TCTGTTCAGATTGGAGCTGATGCTACTTACTTCATGGTGGTTATGGGTGGTTGGTTGAAGTTCACAATTT
CCAACAACGGGAGAACTTTCCGTAACGACCAAGATCGAAATACAGTACCTGAGCAAATCTTAGTTCG
AAACTAATAATTGGTAAATCACAAGGAAAGACGTGTAGTCCACGGATGGACTCTCAAGGAGGTACAA
GGTGCTATCATTAGACTTTAACAACGAATTGATA

Figure S7. Sequences of g-blocks used in this study.

References

1. Baba, T.; Ara, T.; Hasegawa, M.; Takai, Y.; Okumura, Y.; Baba, M.; Datsenko, K.A.; Tomita, M.; Wanner, B.L.; Mori, H. Construction of *Escherichia coli* K-12 in-frame, single-gene knockout mutants: The Keio collection. *Mol. Syst. Biol.* **2006**, *2*, doi:10.1038/msb4100050.
2. Blattner, F.R.; Plunkett, G.; Bloch, C.A.; Perna, N.T.; Burland, V.; Riley, M.; Collado-Vides, J.; Glasner, J.D.; Rode, C.K.; Mayhew, G.F.; et al. The complete genome sequence of *Escherichia coli* K-12. *Science* **1997**, *277*, 1453–1462.
3. Invitrogen BL2-AI ™ Competent Cells–maximum expression with tightest regulation. *Invitrogen.* Carlsbad, CA, USA, **2002**. Accessed on June. 14, 2017. [Online]. Available: http://tools.thermofisher.com/content/sfs/brochures/712_021252_BL21AI_bro.pdf
4. Studier, F.W.; Daegelen, P.; Lenski, R.E.; Maslov, S.; Kim, J.F. Understanding the Differences between Genome Sequences of *Escherichia coli* B Strains REL606 and BL21(DE3) and Comparison of the *E. coli* B and K-12 Genomes. *J. Mol. Biol.* **2009**, *394*, 653–680.
5. Jeong, H.; Barbe, V.; Lee, C.H.; Vallenet, D.; Yu, D.S.; Choi, S.H.; Couloux, A.; Lee, S.W.; Yoon, S.H.; Cattolico, L.; et al. Genome Sequences of *Escherichia coli* B strains REL606 and BL21(DE3). *J. Mol. Biol.* **2009**, *394*, 644–652.
6. Brouns, S.J.J.; Jore, M.M.; Lundgren, M.; Westra, E.R.; Slijkhuis, R.J.H.; Snijders, A.P.L.; Dickman, M.J.; Makarova, K.S.; Koonin, E.V.; van der Oost, J. Small CRISPR RNAs guide antiviral defense in prokaryotes. *Science* **2008**, *321*, 960–964.
